# Supplementary material for: A conserved membrane curvature-generating protein is crucial for autophagosome formation in fission yeast
Source: Nat Commun. 2023 Aug 8;14:4765. doi: 10.1038/s41467-023-40530-4 (PMC10409813; doi:10.1038/s41467-023-40530-4)
Supplement: Supplementary file 1 — Supplementary information [file 41467_2023_40530_MOESM1_ESM.pdf]

Supplementary Information for

A conserved membrane curvature-generating protein is crucial for autophagosome formation in fission yeast

Ning Wang[1], Yoko Shibata[1], Joao A. Paulo[2], Steven P. Gygi[2], and Tom A. Rapoport[1]*

[1]Howard Hughes Medical Institute and Department of Cell Biology, Harvard Medical School, 240 Longwood Avenue, Boston, Massachusetts 02115, USA

[2]Department of Cell Biology, Harvard Medical School, 240 Longwood Avenue, Boston, Massachusetts 02115, USA

* Corresponding author (email: tom_rapoport@hms.harvard.edu)

Supplementary Figure S1

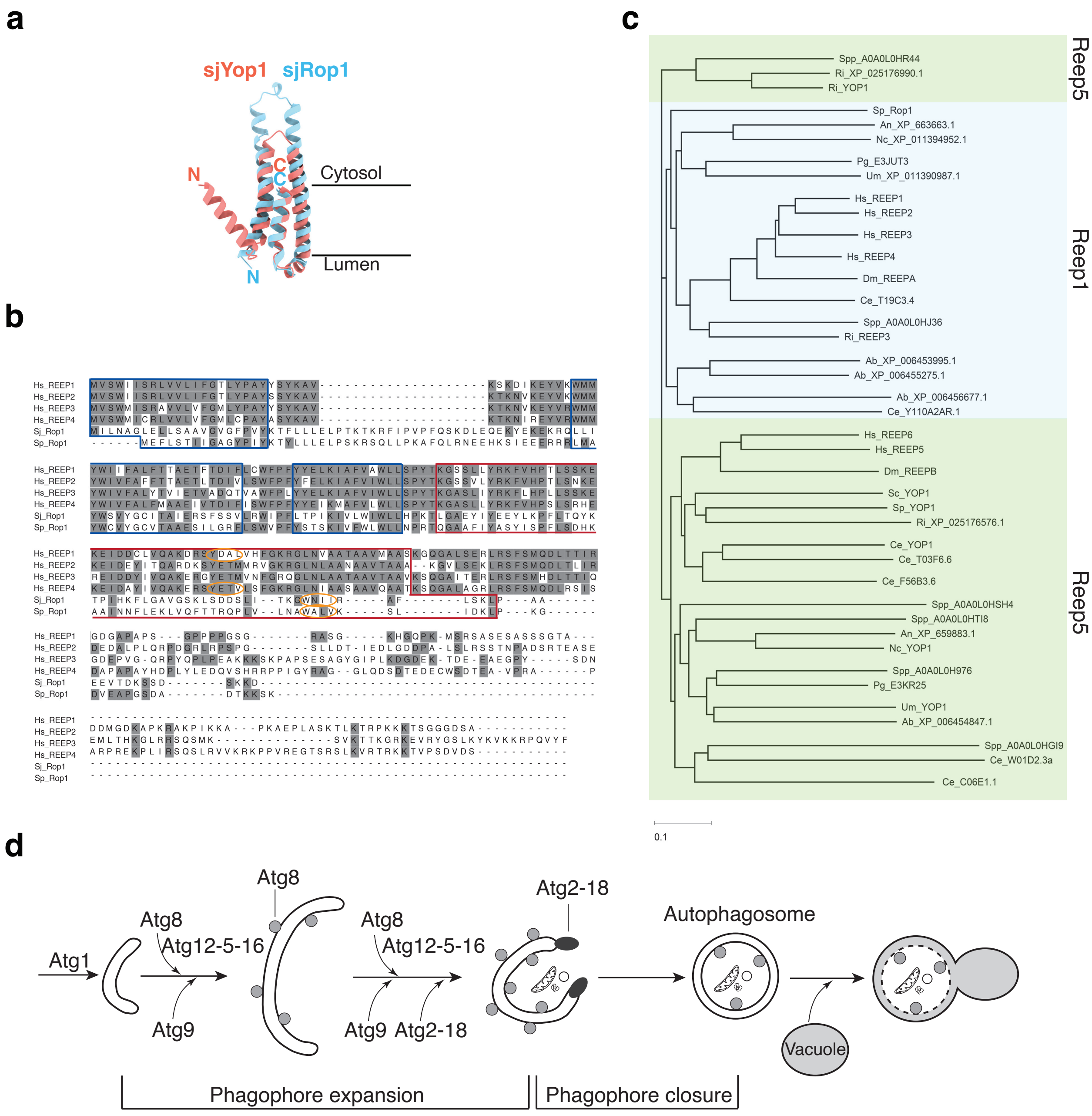

Supplementary Figure S1. Rop1 belongs to a sub-family of the REEPs.
a, Side views of superimposed cartoon models of *S. japonicus* (sj) Rop1 and Yop1, predicted by Alphafold (ref. [7]). The APHs of both proteins were omitted for clarity. **b**, Sequence alignment of *S. pombe* and *S. japonicus* Rop1 with human REEP1-4 proteins using ClustalW. Identical residues are highlighted in gray. Predicted TM, APH, and AIM regions are outlined in blue, red, and orange, respectively. **c**, The phylogeny of REEP proteins was analyzed using ClustalW. Unique gene-encoded REEP protein sequences from representative species of all fungal phyla (Sp, *S. pombe*; Sc, *S. cerevisiae*; Ri, *R. irregularis*; Nc, *N. crassa*; An, *A. nidulans*; Um, *U. maydis*; Ab, *A.bisphorus*; Spp, *S. punctaetus*; Pg, *P. graminis*) were compared with REEP proteins from human (Hs), *D. melanogaster* (Dm), and *C. elegans* (Ce). The REEP5/Yop1 and REEP1 subfamilies are indicated in green and blue, respectively. Note that all species, except *S. cerevisiae*, possess at least one distinct REEP1-like protein. **d**, Steps of autophagy in yeast. The process is conserved in mammals.

Supplementary Figure S2

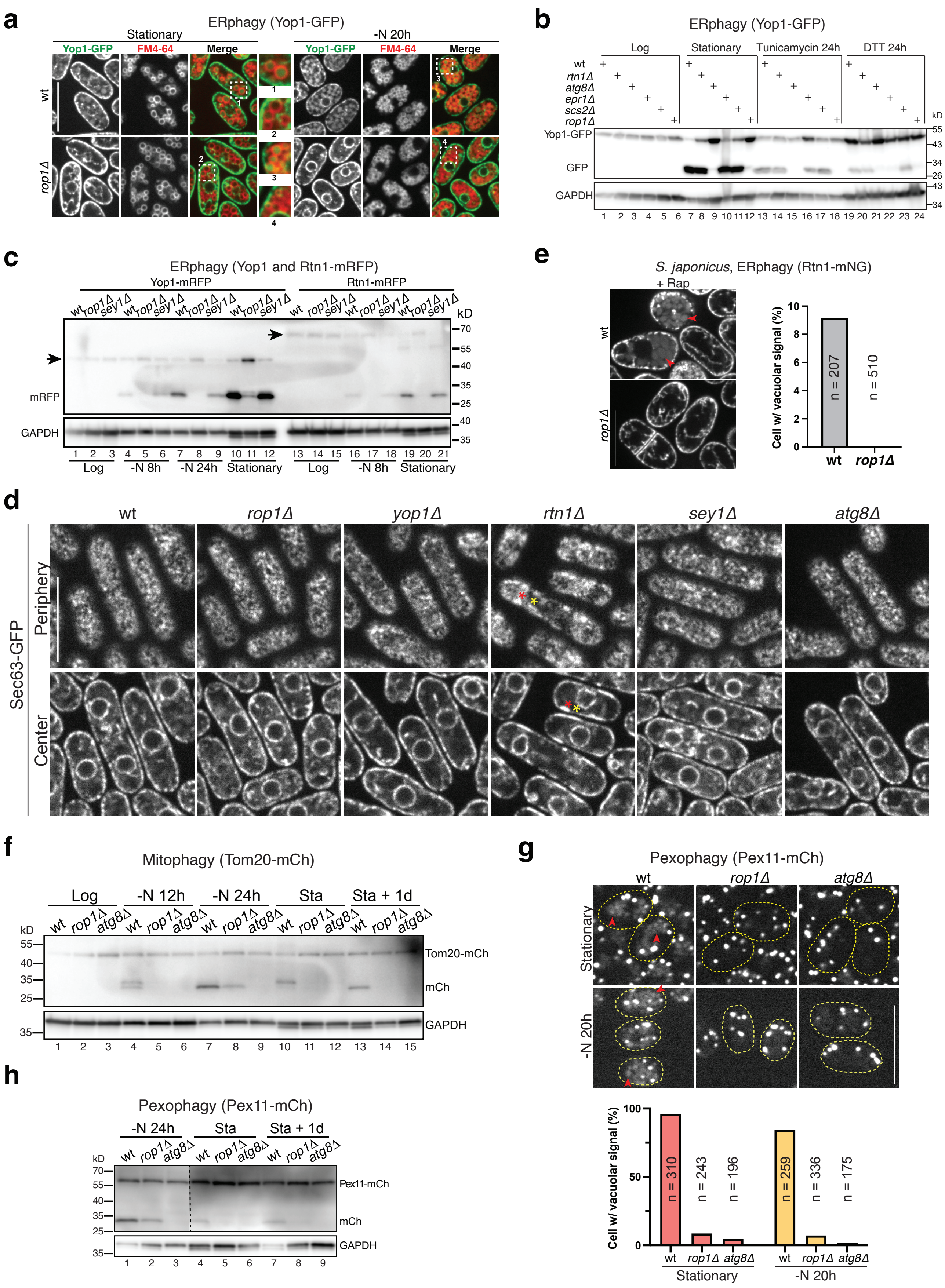

Supplementary Figure S2. Rop1 is involved in the autophagy of organelles.

a, ERphagy was tested with a GFP-fusion of the ER protein Yop1 (Yop1-GFP) expressed at endogenous levels in wild-type (wt) or *rop1Δ S. pombe* cells. Cells in stationary phase or deprived of nitrogen for 20h (-N 20h) were analyzed by fluorescence microscopy. The vacuoles were stained with FM4-64. The right panels show merged images, and boxed regions 1-4 are shown in magnified views. Scale bar, 10 µm. **b**, ERphagy was tested in wt or mutant cells by cleavage of Yop1-GFP. Lysates from cells in logarithmic (Log) or stationary phase, as well as from cells treated with tunicamycin or DTT for 24h, were analyzed by SDS-PAGE and immunoblotting with GFP antibodies. Blotting with GAPDH antibodies served as loading control. Note that Scs2 is a component in the Epr1 pathway[24] and its absence does not affect cleavage of Yop1. **c**, As in **b**, but following ERphagy with Yop1-mRFP or Rtn1-mRFP. Arrows point to the positions of the full-length proteins. **d**, ER morphology in wt and mutant cells. Cells expressing Sec63-GFP were imaged in logarithmic phase by fluorescence microscopy, focusing either on the periphery or center of the cell. Slight ER morphology defects are seen in *rtn1Δ* cells (red asterisks point to abnormal ER accumulation and yellow asterisks to gaps in the cortical ER). **e**, ERphagy was tested in wt or *rop1Δ S. japonicus* cells expressing Rtn1-mNeonGreen (Rtn1-mNG). Autophagy was induced with 200 ng/µl rapamycin in YES medium for 20 h. Arrowheads point to Rtn1-mNG in vacuoles. Scale bar, 10 µm. The right panel shows quantification of the experiment. **f**, Mitophagy was tested with a mCherry fusion of the mitochondrial protein Tom20 (Tom20-mCh) expressed at endogenous levels in wt or mutant cells. The cells were analyzed in Log or stationary (Sta) phase or after nitrogen starvation for different time periods. Some cells were incubated for an additional day after reaching Sta phase (Sta + 1d). In all cases, cell lysates were analyzed by SDS-PAGE and immunoblotting with mCherry antibodies. Blotting with GAPDH antibodies served as loading control. **g**, Pexophagy was tested with a mCherry fusion of the peroxisomal protein Pex11 expressed at endogenous levels in wt and mutant cells. The cells were grown to stationary phase or nitrogen-starved for 20h and analyzed by fluorescence microscopy. Red arrowheads point to Pex11-mCh in vacuoles. The lower panel shows quantification of the experiment. **h**, Pexophagy was tested by cleavage of Pex11-mCh as in **f**.

Supplementary Figure S3

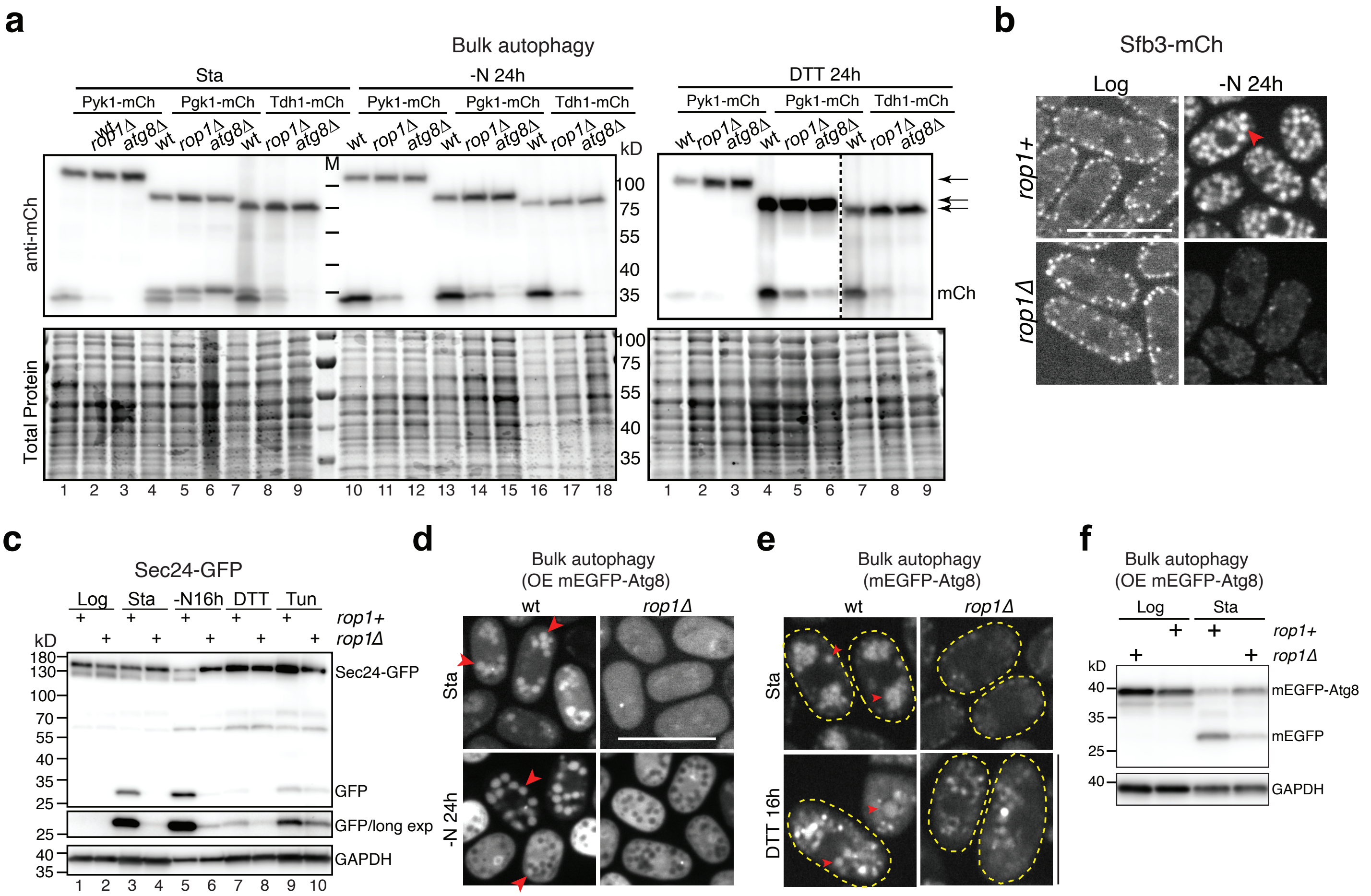

Supplementary Figure S3. Rop1 is involved in bulk autophagy.

a, Vacuolar cleavage was tested with mCherry fusions of the cytosolic proteins Pyk1 (Pyk1-mCh), Pgk1 (Pgk1-mCh), and Tdh1 (Tdh1-mCh). Lysates from cells in stationary (Sta) phase, starved for nitrogen for 24 h, or treated with DTT for 24h, were analyzed by SDS-PAGE and immunoblotting with GFP antibodies. Arrows point to the full-length proteins. As a loading control, the blot was stained for total protein with Revert700 (lower panels). **b**, A mCherry fusion of Sfb3 (Sfb3-mCh), a component of the COPII complex that is found both in the cytosol and bound to ER membranes, was expressed in wt or *rop1*Δ cells. The cells were analyzed in logarithmic (Log) phase or after nitrogen starvation for 24h (-N 24h) by fluorescence microscopy. Red arrowhead points to Sfb3-mCh in vacuoles. Scale bar, 5 µm. **c**, As in **a**, but with a GFP fusion of Sec24 (Sec24-GFP), a component of the COPII complex. Lysates from cells in Log or stationary (Sta) phase, starved for nitrogen for 16 h, or treated with tunicamycin or DTT for 24h, were analyzed by SDS-PAGE and immunoblotting with GFP antibodies. A long exposure (exp) of the immunoblot is shown as well. Blotting with GAPDH antibodies served as loading control. **d**, As in **b**, but for overexpressed mEGFP-tagged Atg8 expressed under the inducible *nmt1* promotor (OE mEGFP-Atg8). Scale bar, 10 µm. **e**, As in **d**, but for mEGFP-Atg8 expressed under its native promotor. The boundaries of the cells are indicated by dotted lines. **f**, As in **a**, but with overexpressed mEGFP-Atg8.

Supplementary Figure S4

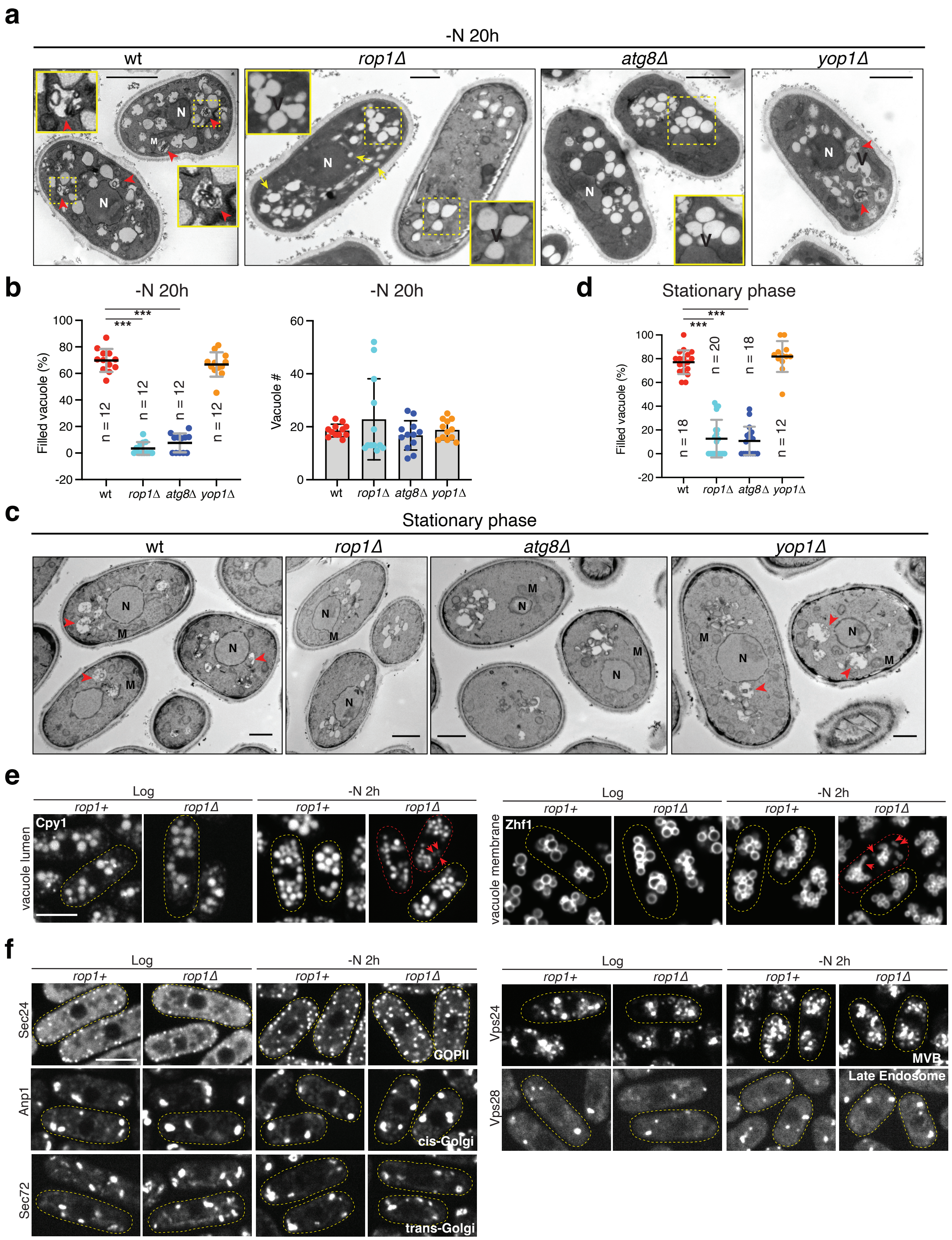

Supplementary Figure S4. Rop1 is involved in autophagosome formation.
a, TEM was performed with nitrogen-starved (-N 20h) wild-type (wt) and mutant cells. Red arrows point to vacuoles filled with electron-dense autophagic cargo, likely residual membranes of autophagosomes. Yellow arrows point to unusually small vacuoles seen in *rop1*Δ cells. N, nucleus; V, vacuole. Scale bar, 1 µm. **b**, Quantification of the results in **a**. Shown is the percentage of filled vacuoles per cell (left panel) and the total number of vacuoles per cell (right panel). Also shown are means and SD. n, number of cells analyzed. *** indicates significant differences with p-values < 0.001, calculated from two-tailed Student t-tests. The exact p-values are listed in the Source Data file. **c**, As in **a**, but for cells in stationary phase. M, mitochondrion. **d**, Quantification of the results in **c**, performed as in **b**. **e**, Markers of the vacuolar lumen (Cpy1) or membrane (Zhf1) were tagged with fluorescent proteins and expressed in *rop1+* or *rop1*Δ cells. The cells were imaged in Log phase or after nitrogen starvation for 2h by confocal fluorescence microscopy. The dotted line shows the boundaries of cells. Red arrowheads point to abnormally small vacuoles. Scale bar, 5 µm. **f**, As in **e**, but with markers of ER exit sites (Sec24), cis-Golgi (Anp1), trans-Golgi (Sec72), multivesicular bodies (Vps24), and late endosomes (Vps28).

Supplementary Figure S5

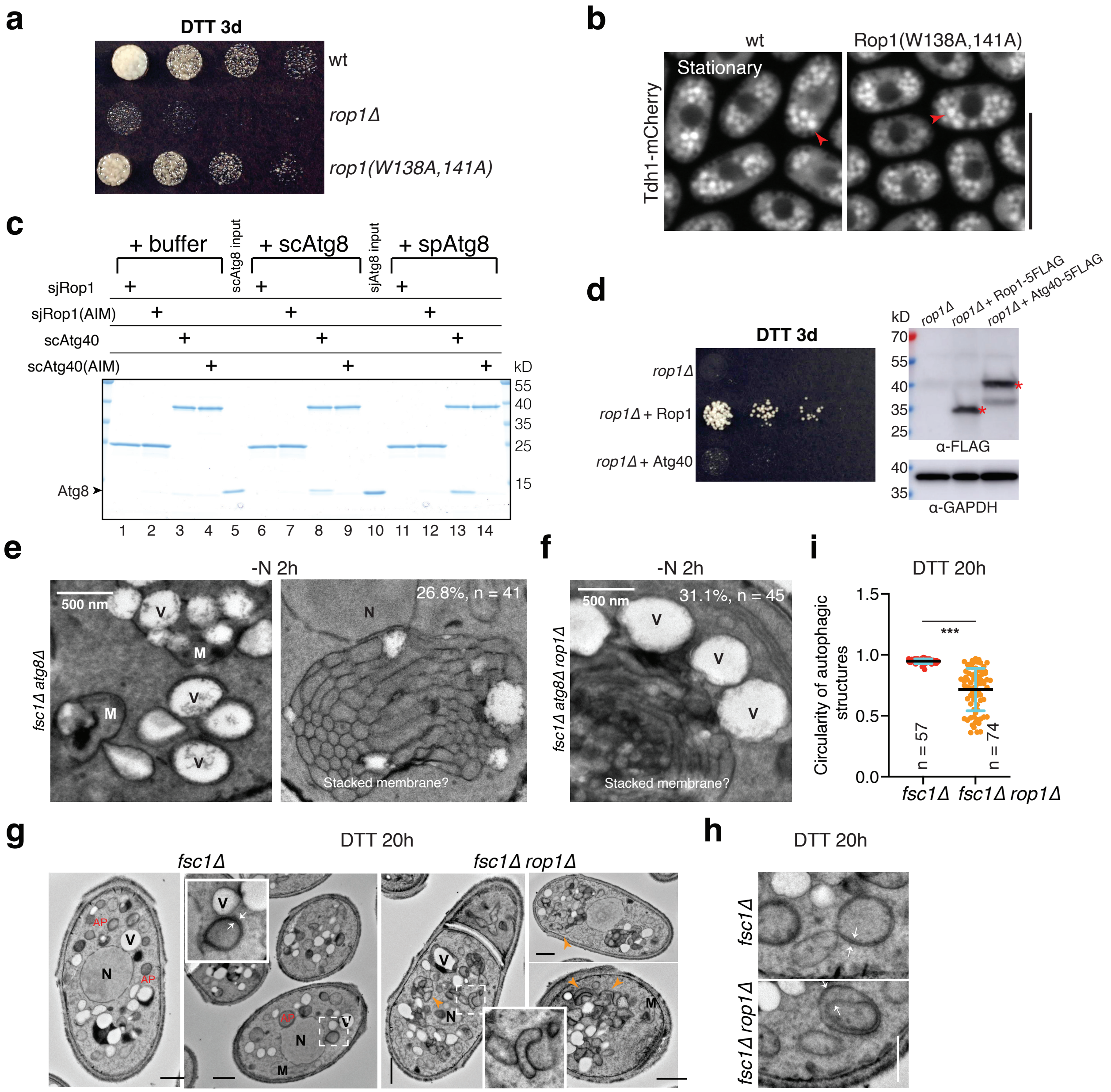

Supplementary Figure S5. Rop1 is not simply an ERphagy receptor.

a, Wild-type (wt) cells or the indicated *rop1* mutant cells were treated with DTT for 3 days and plated after serial dilution. The Rop1 (W138A, 141A) mutant has an abrogated AIM motif. **b**, Bulk autophagy was tested with a mCherry fusion of the cytosolic protein Tdh1 expressed at endogenous levels in wt or *rop1(W138A, 141A)* mutant cells. Stationary phase cells were analyzed by fluorescence microscopy. Red arrowheads point to vacuoles. Scale bar, 10 µm. **c**, *S. japonicus* Rop1 (sjRop1) or *S. cerevisiae* Atg40 (scAtg40), or mutants of these proteins with abrogated AIM motifs [sjRop1(AIM) and scAtg40(AIM)], were tagged with SBP at the C-terminus and purified from *E. coli*. The proteins were incubated with buffer or purified Atg8 from *S. cerevisiae* (scAtg8) or *S. pombe* (spAtg8). The samples were then incubated with streptavidin beads and the bound material analyzed by SDS-PAGE and Coomassie-blue staining. The arrowhead points to the position of co-precipitated Atg8. Input corresponds to 5% used in the pull-down experiments. **d**, *S. cerevisiae* Atg40, tagged with five FLAG epitopes (scAtg40-5FLAG), was expressed in *rop1*Δ cells from the endogenous *rop1* locus. Controls were performed with cells expressing spRop1-5FLAG. The cells were treated with DTT for three days (DTT 3d) and plated after serial dilution (left panel). Cell lysates were also analyzed by SDS-PAGE and immunoblotting with FLAG antibodies (right panel). **e**, *fsc1*Δ *atg8*Δ cells were nitrogen starved for 2 h and analyzed by TEM. Many cells have unusual membrane structures (shown on the right) of unknown origin. N, nucleus; M, mitochondrion; V, vacuole. **f**, As in **e**, but for *fsc1*Δ *rop1*Δ *atg8*Δ cells. 31.1% of the cells again contain the unusual membrane structures. **g**, *fsc1*Δ or *fsc1*Δ *rop1*Δ cells were treated with DTT for 20h and analyzed by TEM. AP, autophagosome. Scale bar, 1 µm. Orange arrow heads point to irregular autophagic structures. Squares outlined with dashed lines are magnified in the insets. White arrows point to autophagosomes with distinct lipid bilayers. **h**, As in **g**, showing additional examples of magnified views of autophagic structures. Scale bar, 500 nm. **i**, Quantification of the circularity of autophagic structures shown in **g**. Also shown are means and SD. n, number of cells analyzed. *** indicates a significant difference with p-value 4.4 x 10^{-18}, calculated from two-sample t-test with unequal variance.

Supplementary Figure S6

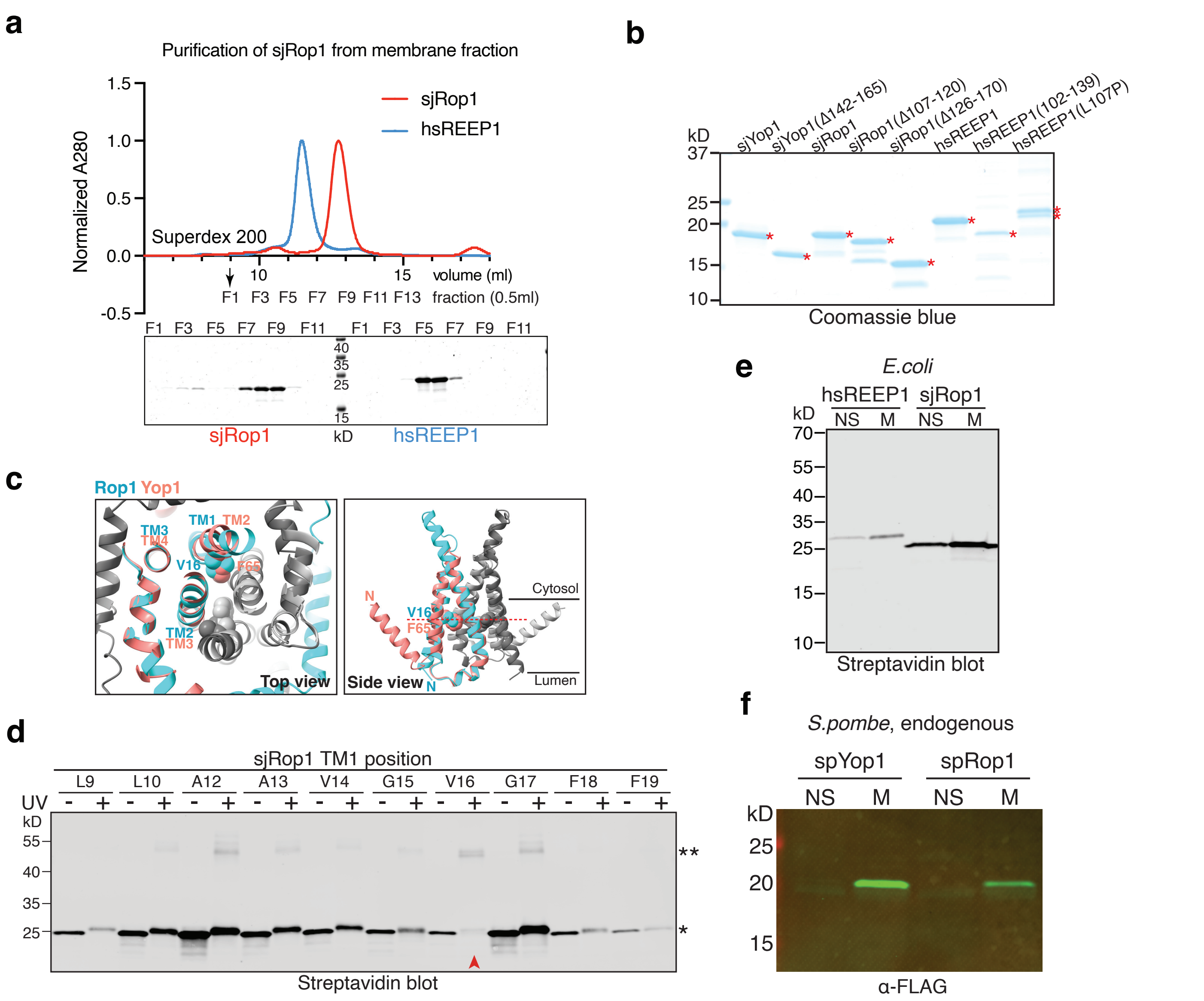

Supplementary Figure S6. Purified Rop1 and hsREEP1 form dimers.

a, SBP-tagged *S. japonicus* Rop1 (sjRop1) or human REEP1 (hsREEP1) were expressed in *E. coli*. A membrane fraction was obtained by ultracentrifugation and solubilized in DDM. The proteins were purified with streptavidin beads and subjected to gel filtration on a Superdex 200 column. The UV absorbance at 280 nm was monitored (upper panel), and fractions were analyzed by SDS-PAGE and Coomassie-blue staining (lower panels). **b**, Wild-type sjRop1 or hsREEP1, or the indicated mutants, were purified and analyzed by SDS-PAGE and Coomassie blue staining. Red stars indicate the positions of the purified proteins. Additional bands are likely caused by proteolysis. **c**, Predicted interaction of the monomers of sjRop1 and sjYop1 in the dimers. Shown is the superposition of the TM segments of the two structures in top (cytosolic) and side views, with one monomer in color (Rop1 in blue and Yop1 in pink) and the other in grey. Amino acids V16 of sjRop1 and F65 of Yop1 give the strongest dimer crosslinks (see panel **d**) and are shown as balls. **d**, SBP-tagged sjRop1 was expressed in *E. coli* with photoreactive Bpa probes incorporated at the indicated positions of TM1 by amber codon suppression. Where indicated, a membrane fraction was irradiated with UV light, and the samples were analyzed by SDS-PAGE, followed by blotting with dye-labeled streptavidin and fluorescence scanning. The red arrowhead indicates the position with strongest dimer crosslinks. **e**, SBP-tagged hsREEP1 and sjRop1 were expressed in *E. coli*. Cell lysates were subjected to ultracentrifugation and the membrane (M) and non-sedimentable (NS) fractions analyzed by SDS-PAGE, followed by blotting with dye-labeled streptavidin and fluorescence scanning. **f**, Yop1 or Rop1 were expressed at endogenous levels in *S. pombe* as FLAG-tagged proteins. Cell lysates were centrifuged as in **e**, and the M and NS fractions analyzed by SDS-PAGE and blotting with FLAG antibodies. Note that both proteins are primarily in the membrane fraction, rather than in non-sedimentable lipoprotein particles.

Supplementary Figure S7

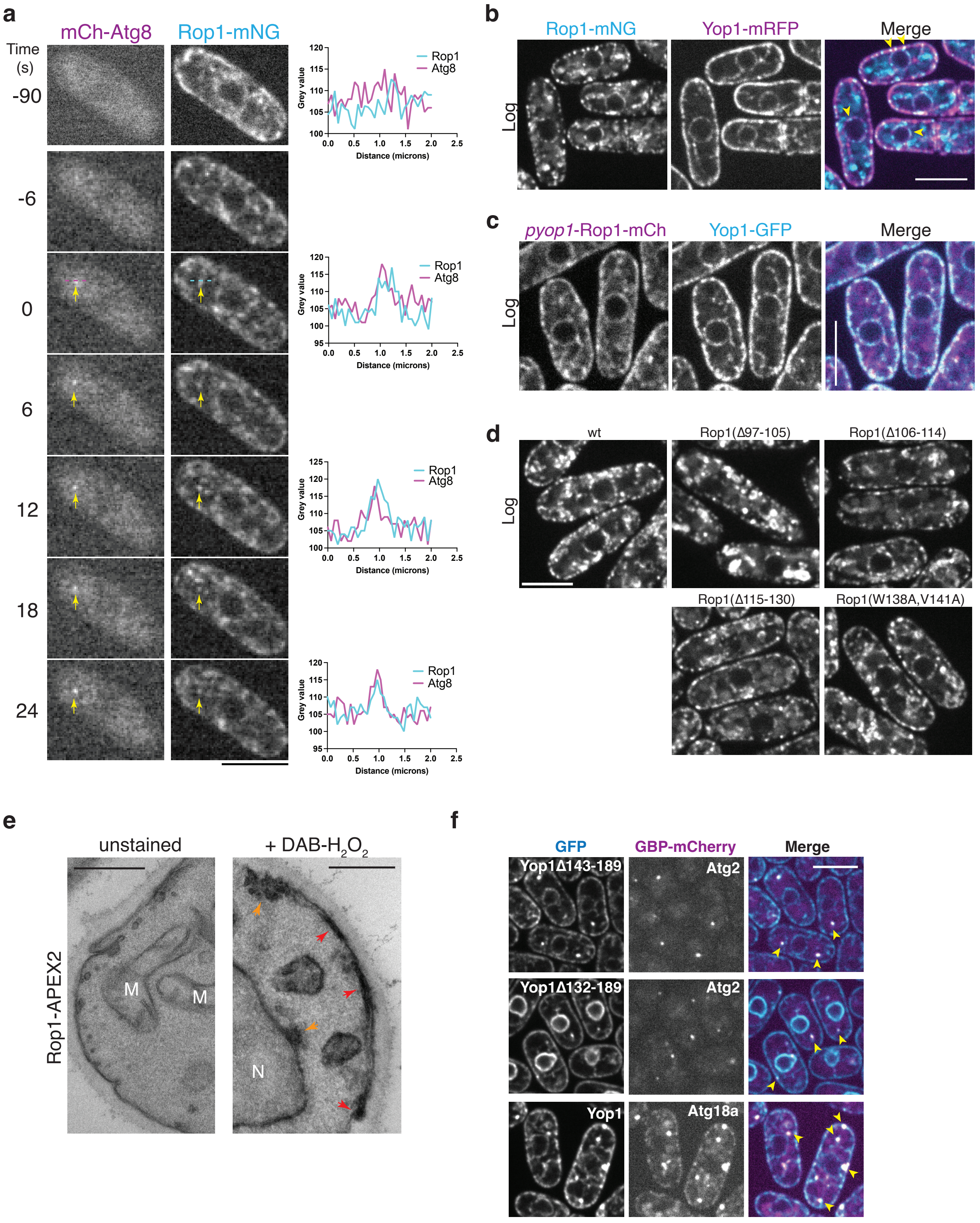

Supplementary Figure S7. Localization of Rop1 in *S. pombe*.
a, Rop1 tagged with mNeonGreen (Rop1-mNG) was co-expressed with mCherry-tagged Atg8 (mCh-Atg8) in *S. pombe* cells. The cells were nitrogen-starved for 3h (-N 3h) and analyzed by confocal fluorescence microscopy. Shown is the montage of a time-lapse movie. At time point zero, a mCh-Atg8 punctum (phagophore) appears (arrow). The right panels show line scans across the Atg8 punctum. Scale bar, 5 µm. **b**, Cells expressing Rop1-mNG and mRFP-tagged Yop1 (Yop1-mRFP) at endogenous levels were visualized in logarithmic (Log) phase. Note that some Rop1 punctae co-localize with Yop1 on the ER (arrowheads). Scale bar, 5 µm. **c**, As in **b**, but Rop1-mCherry was overexpressed under the *yop1* promoter from the *leu1* genomic locus (*pyop1*-Rop1-mCh) while a GFP-fusion of Yop1 (Yop1-GFP) was expressed from its genomic locus. Note that most of the Rop1-mCh molecules now localize with Yop1 throughout the ER. **d**, Cells expressing mNG-tagged wild-type (wt) Rop1 or the indicated mutant Rop1 at the endogenous level were visualized in Log phase. Scale bar, 5 µm. **e**, Cells expressing Rop1-APEX2 were nitrogen-starved for 2h, fixed, and stained without (left) or with (right) DAB and H_2O_2 for 20 min before preparation for TEM. M, mitochondria; N, nucleus. Red arrowheads point to staining of the peripheral ER and orange arrowheads to staining of vesicles near the ER and nuclear envelope. Scale bar, 500 nm. **f**, Atg2 or Atg18a were tagged with GBP-mCherry and co-expressed with mutant or wild-type Yop1-GFP (see also **Fig. 7b**). The cells were imaged after 2h of nitrogen starvation. The arrowheads point to colocalization between the Atg proteins and Yop1. The Yop1Δ143-189 and Yop1Δ132-189 mutants contain or lack the APH, respectively. Scale bar, 5 µm.

Supplementary Figure S8

a

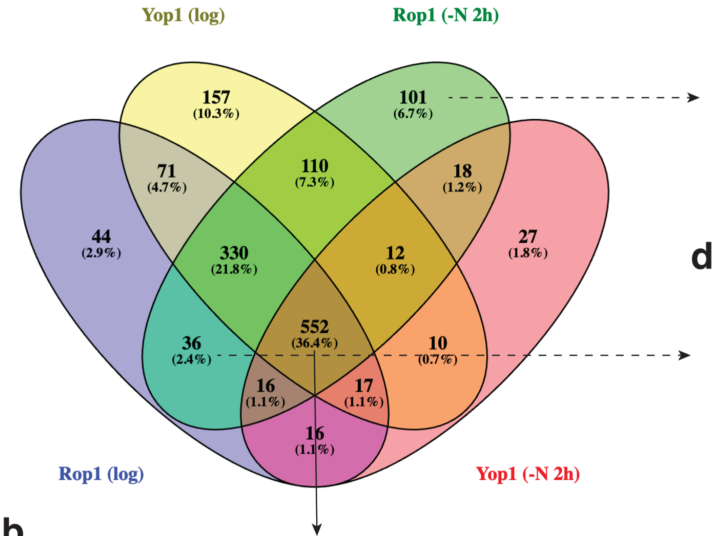

b

Shared by all samples

	Yop1 (log)	Yop1 (-N 2h)	Rop1 (log)	Rop1 (-N 2h)
Fas1 (cytosol)	112	125	96	113
Bip1 (ER)	31	36	35	37
Erg5 (ER)	23	13	23	18
Ost1 (ER)	15	21	15	20
Tom70 (MOM)	32	30	27	31
Tom40 (MOM)	16	5	10	11
Tom20 (MOM)	6	4	7	11
**Yop1**	**11**	**6**	1	0
**Rop1**	0	0	**9**	**8**

c

Associated with Rop1 in starved cells

	Yop1 (log)	Yop1 (-N 2h)	Rop1 (log)	Rop1 (-N 2h)
Abc3	0	0	0	10
Hem14	1	0	0	6
Ish1	1	0	0	5
Cwf3	2	0	1	5
Irc3	2	0	0	5
SPBC1683.05	2	1	0	5
SPBC9B6.07	2	1	1	5
cbp6	0	1	2	5

d

Associated with Rop1 but not Yop1

	Yop1 (log)	Yop1 (-N 2h)	Rop1 (log)	Rop1 (-N 2h)
**Rop1**	0	0	**9**	**8**
SPAC2G11.14	2	1	9	9
Isg1	2	0	9	4
Snz1	2	0	6	3
Cct7	2	1	5	13
SPAC57A10.07	2	1	5	3
Pus1	2	0	5	3
SPAC29E6.06c	1	0	5	5
Prp17	1	1	5	4
Ivn1	2	1	5	5
Cdk9	2	2	5	7
Rgf1	1	0	4	4
Hal4	1	0	3	4
Plh1	2	0	3	4
SPBC18E5.01	1	0	3	5

e

Autophagy protein identified in pull-down

	Yop1 (log)	Yop1 (-N 2h)	Rop1 (log)	Rop1 (-N 2h)
**Atg9**	3	0	1	2

Supplementary Figure S8. Search for interaction partners of Rop1.
a, FLAG-tagged Rop1 or Yop1 (Rop1-FLAG or Yop1-FLAG) were expressed at endogenous levels in cells grown in rich medium to logarithmic phase or in nitrogen-depleted medium for 2h. A membrane fraction was isolated from cell lysates, solubilized in DDM, and subjected to immunoprecipitation with FLAG antibodies. Co-precipitated proteins were analyzed by mass spectrometry. Shown is a Venn diagram for the number of proteins identified in the four different pull-down experiments. Note that most proteins were pulled down by both Rop1-FLAG and Yop1-FLAG. **b**, Examples of proteins found in all four pull-down experiments. The table gives the number of peptides detected for each protein. **c**, Examples of proteins exclusively associated with Rop1 in starved cells. **d**, Examples of proteins associated primarily with Rop1, but not Yop1. **e**, Among autophagy components, only the integral membrane protein Atg9 was detected. The low number of peptides and the fact that these were found with both Rop1 and Yop1 pull-downs, suggests that Atg9 was non-specifically associated with these proteins. The raw data is provided in the Source Data file.

Supplementary Table 1. *S. pombe* strains used in this study.

Name	Genotype
Sp1	*S. pombe 972 h⁻*
Sp2	*h⁺ ade6-M216 leu1-32*
Sp7	*h⁺ rop1Δ::natMX6 ade6-M216 leu1-32*
Sp11	*h⁺ yop1-tdTomato-natMX6 ade6-M216 leu1-32*
Sp19	*h⁺ yop1-GFP-natMX ade-M216 leu1-32*
Sp21	*rtn1Δ::kanMX yop1-GFP-natMX ade6 leu1-32*
Sp22	*rop1Δ::kanMX6 yop1-tdTomato-natMX6 ade6-M216 leu1-32*
Sp25	*rop1Δ::kanMX6 yop1-GFP-natMX ade-M216 leu1-32*
Sp26	*epr1Δ::kanMX6 yop1-GFP-natMX ade-M216 leu1-32*
Sp27	*atg8Δ::kanMX6 yop1-GFP-natMX ade-M216 leu1-32*
Sp28	*scs2Δ::kanMX6 yop1-GFP-natMX ade-M216 leu1-32*
Sp32	*h⁻ atg8Δ::natMX6 ade6-M210 leu1-32*
Sp33	*h⁺ kanMX6-pnmt1-mEGFP-atg8*
Sp35	*epr1Δ::natMX6 yop1-tdTomato-natMX6 ade6 leu1-32*
Sp36	*atg8Δ::natMX6 yop1-tdTomato-natMX6 ade6 leu1-32*
Sp38	*h⁻ rop1-5FLAG-kanMX6 ade6-M210 leu1-32*
Sp41	*rop1Δ::kanMX6 kanMX6-pnmt1-mEGFP-atg8*
Sp43	*h⁺ yop1Δ::kanMX ade-M216 leu1-32*
Sp52	*h⁻ rop1(1-97)-SBP-kanMX6 ade6-M210 leu1-32*
Sp53	*h⁻ rop1(1-123)-SBP-kanMX6 ade6-M210 leu1-32*
Sp69	*h⁻ rop1(1-147)-SBP-kanMX6 ade6-M210 leu1-32*
Sp71	*h⁻ kanMX6-patg8-mEGFP-atg8 ade6-M210 leu1-32*
Sp81	*h⁻ rop1(W138A,V141A)-SBP-kanMX6 ade6-M210 leu1-32*
Sp82	*h⁺ sec24-GFP-ura4⁺ ade6-M216 leu1-32 ura4-D18*
Sp84	*h⁺ anp1-GFP-ura4 ade6-216 leu1-32 ura4-D18*
Sp86	*h⁺ sec72-GFP-ura4 ade6-216 leu1-32 ura4-D18*
Sp92	*rop1Δ::natMX6 kanMX6-patg8-mEGFP-atg8 ade6 leu1-32*
Sp97	*h⁻ pnmt1-YFP-leu1⁺ ade6 leu1-32*
Sp99	*h⁻ rop1Δ::natMX6 pnmt1-YFP-leu1⁺ ade6 leu1-32*
Sp124	*rop1Δ::natMX6 sec24-GFP-ura4⁺ ade6-M216 leu1-32 ura4-D18*
Sp124	*rop1Δ::natMX6 sec24-GFP-ura4 ade6-M216 leu1-32 ura4-D18*
Sp129	*rop1Δ::natMX6 sec72-GFP-ura4 ade6-M216 ura4-D18 leu1-32*
Sp138	*rtn1-mRFP-kanMX6 ade6-M216 leu1-32*
Sp139	*rop1Δ::natMX6 rtn1-mRFP-kanMX6 ade6-M216 leu1-32*
Sp141	*sey1Δ::natMX rtn1-mRFP-kanMX6 ade6-M216 leu1-32*
Sp143	*yop1-mRFP-kanMX6 ade6-M216 leu1-32*
Sp144	*rop1Δ::natMX6 yop1-mRFP-kanMX6 ade6-M216 leu1-32*
Sp146	*sey1Δ::natMX yop1-mRFP-kanMX6 ade6-M216 leu1-32*
Sp149	*h⁺ fsc1Δ::hphMX ade6-M216 leu1-32 ura4-D18*
Sp151	*h⁻ fsc1Δ::hphMX rop1Δ::natMX6 ade6-M216 leu1-32 ura4-D18*
Sp152	*h⁻ cpy1-mNeonGreen-hphMX6 ade6-M210 leu1-32 ura4-D18*
Sp157	*rop1-mNeonGreen-natMX6 yop1-mRFP-kanMX6 ade6-M216 leu1-32*
Sp162	*kanMX6-pnmt1-mEGFP-atg8 fsc1Δ::hphMX ade6-M216 leu1-32 ura4-D18*

Sp163	*kanMX6-pnmt1-mEGFP-atg8 fsc1Δ::hphMX rop1Δ::natMX6 ade6-M216 leu1-32 ura4-D18*
Sp170	*cpy1-mNeonGreen-hphMX6 rop1Δ::natMX6 ade6-M210 leu1-32 ura4-D18*
Sp175	h^{+} *rop1(Δ133-147)-SBP-kanMX6 ade6-M216 leu1-32*
Sp197	h^{+} *vps24-mEGFP-hphMX6 ade6-M210 leu1-32 ura4-D18*
Sp198	h^{+} *vps28-mEGFP-hphMX6 ade6-M210 leu1-32 ura4-D18*
Sp208	*sfb3-mCherry-hphMX6 ade6 leu1-32*
Sp209	*rop1Δ::natMX6 sfb3-mCherry-hphMX6 ade6 leu1-32*
Sp228	h^{-} *rop1(Δ97-105)-SBP-kanMX6 ade6-M210 leu1-32*
Sp229	h^{-} *rop1(Δ106-114)-SBP-kanMX6 ade6-M210 leu1-32*
Sp230	h^{-} *rop1(Δ166-181)-SBP-kanMX6 ade6-M210 leu1-32*
Sp245	*tom20-mCherry-hphMX6 ade6 leu1-32*
Sp246	*rop1Δ::natMX6 tom20-mCherry-hphMX6 ade6 leu1-32*
Sp248	*atg8Δ::kanMX6 tom20-mCherry-hphMX6 ade6 leu1-32*
Sp265	h^{+} *tdh1-mCherry-hphMX6 ade6 leu1-32*
Sp266	*rop1Δ::natMX6 tdh1-mCherry-hphMX6 ade6 leu1-32*
Sp267	*atg8Δ::kanMX6 tdh1-mCherry-hphMX6 ade6 leu1-32*
Sp269	h^{-} *pyk1-mCherry-hphMX6*
Sp270	h^{-} *pgk1-mCherry-hphMX6*
Sp272	h^{-} *hsc1-GFP-hphMX ade6-M210 leu1-32*
Sp274	*rop1Δ::natMX6*
Sp275	*atg8Δ::natMX6*
Sp276	*yop1Δ::kanMX*
Sp278	*pgk1-mCherry-hphMX6 rop1Δ::natMX6*
Sp279	*pgk1-mCherry-hphMX6 atg8Δ::kanMX6*
Sp280	*hsc1-GFP-hphMX rop1Δ::natMX6 ade6-M210 leu1-32*
Sp281	*hsc1-GFP-hphMX atg8Δ::kanMX6 ade6-M210 leu1-32*
Sp282	h^{-} *pex11-mCherry-hphMX6*
Sp285	*pyk1-mCherry-hphMX6 rop1Δ::natMX6*
Sp286	*pyk1-mCherry-hphMX6 atg8Δ::kanMX6*
Sp293	*kanMX6-p41nmt1-mCherry-atg8 kanMX6-p81nmt1-rop1-mNeonGreen-natMX6 ade6 leu1-32*
Sp294	*rop1Δ::natMX6 pex11-mCherry-hphMX6*
Sp295	*atg8Δ::kanMX6 pex11-mCherry-hphMX6 ade6*
Sp298	h^{-} *rop1Δ::prop1-atg40-5FLAG-kanMX6 ade6-M210 leu1-32*
Sp336	*atg2-GBP-mcherry-kanMX6 yop1-GFP-natMX leu1-32*
Sp356	h^{+} *sec63-GFP-natMX*
Sp358	*atg2-tdTomato-hphMX6 kanMX6-patg8-mEGFP-atg8 ade6-M216 leu1-32*
Sp359	*rop1Δ::natMX6 atg2-tdTomato-hphMX6 kanMX6-patg8-mEGFP-atg8 ade6-M216 leu1-32*
Sp364	*rop1Δ::hphMX atg2-GBP-mcherry-kanMX6 yop1-GFP-natMX*
Sp370	*rop1Δ::natMX6 anp1-GFP-ura4 ade6-216 leu1-32 ura4-D18*
Sp378	*rop1Δ::kanMX6 sec63-GFP-natMX*
Sp380	*rop1Δ::hphMX atg2-GBP-mcherry-kanMX6 sec63-GFP-natMX*
Sp391	*rop1Δ::hphMX yop1-GFP-natMX atg18a-GBP-mcherry-kanMX6 leu1-32*
Sp392	*rop1Δ::hphMX yop1-GFP-natMX atg18b-GBP-mcherry-kanMX6 ade6-M216*
Sp394	*rop1Δ::natMX6 vps24-mEGFP-hphMX6 ade6-M210 leu1-32 ura4-D18*
Sp395	*rop1Δ::natMX6 vps28-mEGFP-hphMX6 ade6-M210 leu1-32 ura4-D18*

Sp404	*rop1Δ::hphMX atg2-GBP-mcherry-kanMX6 yop1(d132-189)-GFP-natMX*
Sp405	*rop1Δ::hphMX atg2-GBP-mcherry-kanMX6 yop1(d143-189)-GFP-natMX*
Sp411	*h^+ zhf1-mNeonGreen-kanMX6 ade6-M216 leu1-32*
Sp412	*atg1-tdTomato-hphMX6 kanMX6-patg8-mEGFP-atg8*
Sp413	*rop1Δ::natMX6 atg1-tdTomato-hphMX6 kanMX6-patg8-mEGFP-atg8*
Sp418	*atg9-tdTomato-hphMX6 kanMX6-patg8-mEGFP-atg8 ade6-M216 leu1-32*
Sp424	*rop1Δ::natMX6 atg9-tdTomato-hphMX6 kanMX6-patg8-mEGFP-atg8 leu1-32*
Sp434	*rop1Δ::natMX6 zhf1-mNeonGreen-kanMX6 ade6-M216 leu1-32*
Sp445	*h^- kanMX6-patg8-mEGFP-atg8 atg5-mcherry-hphMX6 ade6-M210 leu1-32*
Sp446	*h^- rop1Δ::natMX6 kanMX6-patg8-mEGFP-atg8 atg5-mcherry-hphMX6 ade6 leu1-32*
Sp454	*pyop1-rop1-mcherry-leu1 yop1-GFP-natMX ade6 leu1-32*
Sp458	*atg2-GBP-hphMX6 rop1Δ::kanMX6 yop1-GFP-natMX tdh1-mCherry-hph ade6-M21X*
Sp459	*rop1Δ::kanMX6 yop1-GFP-natMX tdh1-mCherry-hph ade6-M21X*
Sp460	*atg2-GBP-hphMX6 rop1Δ::kanMX6 sec63-GFP-natMX tdh1-mCherry-hph ade6-M21X*
Sp461	*atg18b-GBP-hphMX6 rop1Δ::kanMX6 sec63-GFP-natMX tdh1-mCherry-hph ade6-M21X*
Sp471	*h^+ rop1-APEX2-flag-natMX6 ade6-M216 leu1-32*
Sp473	*h^+ atg2-APEX2-flag-natMX6 ade6-M216 leu1-32*
Sp474	*h^+ atg2-APEX2-flag-natMX6 ade6-M216 leu1-32*
Sp502	*hsc1-GFP-hphMX rop1(Δ97-105)-SBP-kanMX6 ade6-M21X leu1-32*
Sp503	*h^+ hsc1-GFP-hphMX ade6-M210 leu1-32*
Sp504	*hsc1-GFP-hphMX rop1(Δ106-114)-SBP-kanMX6 ade6-M21X leu1-32*
Sp505	*hsc1-GFP-hphMX rop1(Δ115-130)-SBP-kanMX6 ade6-M21X leu1-32*
Sp506	*hsc1-GFP-hphMX rop1(W138A, V141A)-SBP-kanMX6 ade6-M21X leu1-32*
Sp507	*h^+ atg2Δ::kanMX6 ade6-M216 leu1-32*
Sp508	*h^+ atg8Δ::natMX6 ade6-M216 leu1-32*
Sp514	*h^- atg8Δ:natMX6 fsc1Δ::hphMX rop1Δ::kanMX6 ade6-M210 leu1-32*
Sp515	*h^- atg8Δ:natMX6 fsc1Δ::hphMX ade6-M210 leu1-32*
Sp516	*h^+ rtn1Δ::hphMX sec63-GFP-natMX ade-M216 leu1-32*
Sp517	*h^+ yop1Δ::kanMX sec63-GFP-natMX ade-M216 leu1-32*
Sp518	*h^- atg8Δ:natMX6 sec63-GFP-kanMX ade6-M210 leu1-32*
Sp519	*h^+ sey1Δ::natMX sec63-GFP-kanMX ade6-M216 leu1-32*
Sp524	*h^+ yop1-flag-kanMX ade6-M216 leu1-32*
Sp525	*h^+ rop1-flag-kanMX ade6-M216 leu1-32*

Supplementary Table 2. Plasmids used to express proteins in *E. coli* and *S. pombe*.

Name		Note
NWP385	pET21b-sjRop1-3C-SBP	Express sjRop1 in *E. coli*
NWP390	pET21b-sjRop1(Δ126-170)-3C-SBP	Express sjRop1 mutant in *E. coli*
NWP391	pET21b-sjRop1(Δ107-120)-3C-SBP	Express sjRop1 mutant in *E. coli*
NWP516	pET21b-sjRop1(Δ103-118)-3C-SBP	Express sjRop1 mutant in *E. coli*
NWP517	pET21b-sjRop1(Δ120-134)-3C-SBP	Express sjRop1 mutant in *E. coli*
NWP395	pET28-hsREEP1-TEV-SBP	Express human REEP1 in *E. coli*
NWP485	pET28-hsREEP1(L107P)-TEV-SBP	Express human REEP1 mutant in *E. coli*
NWP486	pET28-hsREEP1(Δ102-139)-TEV-SBP	Express human REEP1 mutant in *E. coli*
NWP481	pET21b- sjRop1(V16Bpa)-3C-SBP	V16 codon was mutated to TAG for incorporation of Bpa at this position; Amber codon incorporated at other positions of TM1 are available for request
NWP412	pET21b-His10-TEV-scAtg8(1-116)	Express scAtg8 amino acid 1-116 in *E. coli*
NWP451	pGEX-6p-spAtg8	Express spAtg8 in *E. coli*
NWP410	pET28-scAtg40-TEV-SBP	Express scAtg40 in *E. coli*
NWP419	pET28-scAtg40(Y242A, M245A)-TEV-SBP	Express scAtg40 LIR mutant in *E. coli*
NWP413	pET21b-sjRop1(W144A, I147A)-3C-SBP	Express sjRop1 LIR mutant in *E. coli*
NWP435	pJK148-*nmt1*-EYFP	To integrate and express EYFP (fused with HHGNSGPPPPGAFPHPLEGGDPPVAT at N-terminus) at *leu1* locus in *S. pombe*
NWP436	pFA6a-kanMX6-*patg8*-mEGFP	To express mEGFP N-terminally tagged spAtg8 at its genomic locus
NWP463	Topo-*prop1*-Rop1-5xFLAG-kanMX6	To express Rop1 tagged with 5xFLAG at its genomic locus; deletion or point mutations affecting APH and predicted LIR motif of Rop1 are available for request
NWP504	Topo-*prop1*-Atg40-5FLAG-kanMX6	To express Atg40 tagged with 5xFLAG at Rop1's genomic locus
