# Supplementary material for: A conserved membrane curvature-generating protein is crucial for autophagosome formation in fission yeast
Source: Nat Commun. 2023 Aug 8;14:4765. doi: 10.1038/s41467-023-40530-4 (PMC10409813; doi:10.1038/s41467-023-40530-4)
Supplement: Supplementary file 3 — Reporting Summary [file 41467_2023_40530_MOESM3_ESM.pdf]

nature portfolio

Corresponding author(s): Tom Rapoport

Last updated by author(s): Jul 3, 2023

Reporting Summary

Nature Portfolio wishes to improve the reproducibility of the work that we publish. This form provides structure for consistency and transparency in reporting. For further information on Nature Portfolio policies, see our Editorial Policies and the Editorial Policy Checklist.

Statistics

For all statistical analyses, confirm that the following items are present in the figure legend, table legend, main text, or Methods section.

n/a	Confirmed	
☐	☒	The exact sample size (n) for each experimental group/condition, given as a discrete number and unit of measurement
☐	☒	A statement on whether measurements were taken from distinct samples or whether the same sample was measured repeatedly
☐	☒	The statistical test(s) used AND whether they are one- or two-sided<br>*Only common tests should be described solely by name; describe more complex techniques in the Methods section.*
☐	☒	A description of all covariates tested
☒	☐	A description of any assumptions or corrections, such as tests of normality and adjustment for multiple comparisons
☐	☒	A full description of the statistical parameters including central tendency (e.g. means) or other basic estimates (e.g. regression coefficient) AND variation (e.g. standard deviation) or associated estimates of uncertainty (e.g. confidence intervals)
☐	☒	For null hypothesis testing, the test statistic (e.g. F, t, r) with confidence intervals, effect sizes, degrees of freedom and P value noted<br>*Give P values as exact values whenever suitable.*
☒	☐	For Bayesian analysis, information on the choice of priors and Markov chain Monte Carlo settings
☒	☐	For hierarchical and complex designs, identification of the appropriate level for tests and full reporting of outcomes
☒	☐	Estimates of effect sizes (e.g. Cohen's d, Pearson's r), indicating how they were calculated

Our web collection on statistics for biologists contains articles on many of the points above.

Software and code

Policy information about availability of computer code

Data collection	MetaMorph (version 7.8), Nikon NIS-Elements, ImageQuant, LiCor Acquisition software, AMT imaging software
Data analysis	Fiji(Image J), Microsoft Excel, Graphpad Prism, MSconvert (Comet 2021.01.0)

For manuscripts utilizing custom algorithms or software that are central to the research but not yet described in published literature, software must be made available to editors and reviewers. We strongly encourage code deposition in a community repository (e.g. GitHub). See the Nature Portfolio guidelines for submitting code & software for further information.

Data

Policy information about availability of data

All manuscripts must include a data availability statement. This statement should provide the following information, where applicable:

- Accession codes, unique identifiers, or web links for publicly available datasets
- A description of any restrictions on data availability
- For clinical datasets or third party data, please ensure that the statement adheres to our policy

Data availability
The mass spectrometry proteomics data have been deposited to the ProteomeXchange Consortium via the PRIDE partner repository with the dataset identifier PXD043260. Source data are provided with this paper. Images are available from the corresponding author upon request.

Research involving human participants, their data, or biological material

Policy information about studies with human participants or human data. See also policy information about sex, gender (identity/presentation), and sexual orientation and race, ethnicity and racism.

Reporting on sex and gender	N/A
Reporting on race, ethnicity, or other socially relevant groupings	N/A
Population characteristics	N/A
Recruitment	N/A
Ethics oversight	N/A

Note that full information on the approval of the study protocol must also be provided in the manuscript.

Field-specific reporting

Please select the one below that is the best fit for your research. If you are not sure, read the appropriate sections before making your selection.

[x] Life sciences [ ] Behavioural & social sciences [ ] Ecological, evolutionary & environmental sciences

For a reference copy of the document with all sections, see nature.com/documents/nr-reporting-summary-flat.pdf

Life sciences study design

All studies must disclose on these points even when the disclosure is negative.

Sample size	Power analysis was not performed to predetermine sample size. For yeast EM and confocal imaging, 5-10 images were taken for each sample with each containing sufficient cell amounts based on field standards; experiments were repeated 2-4 times and all the objects in 3-5 randomly-selected representative images were used for quantification.
Data exclusions	No data was excluded from the analyses
Replication	Each experiment was biologically replicated twice or more, unless otherwise noted. All attempts at replication were successful.
Randomization	All starting samples (ie.. cells) were randomly allocated into groups for experimentation. All image fields used for quantification were randomly selected.
Blinding	Investigators were not blinded to group allocation during data collection and analysis. Differences in phenotype were clear amongst different samples/conditions and double blinding would be ineffective.

Reporting for specific materials, systems and methods

We require information from authors about some types of materials, experimental systems and methods used in many studies. Here, indicate whether each material, system or method listed is relevant to your study. If you are not sure if a list item applies to your research, read the appropriate section before selecting a response.

Materials & experimental systems

n/a	Involved in the study	
[ ]	[x]	Antibodies
[x]	[ ]	Eukaryotic cell lines
[x]	[ ]	Palaeontology and archaeology
[x]	[ ]	Animals and other organisms
[x]	[ ]	Clinical data
[x]	[ ]	Dual use research of concern
[x]	[ ]	Plants

Methods

n/a	Involved in the study	
[x]	[ ]	ChIP-seq
[x]	[ ]	Flow cytometry
[x]	[ ]	MRI-based neuroimaging

Antibodies

Antibodies used	antiGFP mouse monoclonal antibody (JL-8, Takara, Cat No. 632381); GFP rabbit polyclonal antibody (ChromoTek, Cat No. pabg1); anti-mCherry mouse monoclonal antibody (1C51, Abcam, Cat No. ab125096); and anti-GAPDH mouse monoclonal antibody (GA1R, Abcam, Cat No. ab125247).
Validation	Primary antibodies were validated by detecting no signal for negative controls and strong and specific signals for positive controls with appropriate size bands by manufacturer. Antibody validation information can be found in manufacturer's websites.
